# Versatile microrobotics using simple modular subunits

**DOI:** 10.1038/srep30472

**Published:** 2016-07-28

**Authors:** U Kei Cheang, Farshad Meshkati, Hoyeon Kim, Kyoungwoo Lee, Henry Chien Fu, Min Jun Kim

**Affiliations:** 1 Dept. of Mechanical Engineering & Mechanics, Drexel University, Philadelphia, PA 19104, USA; 2 Dept. of Mechanical Engineering, University of Nevada, Reno, Reno, NV 89557, USA; 3 Dept. of Mechanical Engineering, Southern Methodist University, Dallas, TX 75275, USA; 4 Dept. of Computer Science, Yonsei University, Seoul, 120-749, Republic of Korea; 5 Dept. of Mechanical Engineering, University of Utah, Salt Lake City, UT 84112, USA

## Abstract

The realization of reconfigurable modular microrobots could aid drug delivery and microsurgery by allowing a single system to navigate diverse environments and perform multiple tasks. So far, microrobotic systems are limited by insufficient versatility; for instance, helical shapes commonly used for magnetic swimmers cannot effectively assemble and disassemble into different size and shapes. Here by using microswimmers with simple geometries constructed of spherical particles, we show how magnetohydrodynamics can be used to assemble and disassemble modular microrobots with different physical characteristics. We develop a mechanistic physical model that we use to improve assembly strategies. Furthermore, we experimentally demonstrate the feasibility of dynamically changing the physical properties of microswimmers through assembly and disassembly in a controlled fluidic environment. Finally, we show that different configurations have different swimming properties by examining swimming speed dependence on configuration size.

Modular microrobotics are attractive since they may allow for much greater versatility, enabling a single system to navigate many different environments or perform multiple tasks, which are key hurdles for *in vivo* biomedical applications. While existing microrobots have demonstrated excellent capabilities to move or work in homogenous environments and to complete a single specific task, modular robots with the ability to change their shape can adapt to varying tasks and environments and can respond to unpredictable situations^1^. This is advantageous for applications that requires the use of micro/nanorobots over a wide range of different environments. Two illustrative examples are targeted/localized drug delivery and minimally invasive surgery.

For drug delivery, recent research in nanomedicine has shown great promise to improve mediated delivery to tumors via the use of nanoparticles^2^,^3^. The success rate of using nanoparticle drug delivery methods depends on many uncontrolled factors in various vastly different environments such as circulation in the blood stream, diffusion inside tumor microenvironments, and biological barriers such as extracellular matrix (ECM)^4^. For example, in drug delivery the inability to overcome these environments can lead to poor drug accumulation on tumor cells^5^. Current approaches in nanoparticle drug delivery focus on using diffusive transport and targeting ligands, but there are also several studies using a microrobotics approach to enhance targeting by using magnetic field gradients for direct manipulation^6^,^7^,^8^ which show the potential of using microrobotics in nanoparticle drug delivery. Nonetheless, the challenge of traversing many different environments remains formidable. Since microorganisms use a vast variety of shapes and propulsion methods to navigate many different bio-environments, reconfigurable microrobots that can take many different shapes and sizes could be useful to tackle the diversity of *in vivo* environments. For example, larger swimmers can move faster according to the Resistive Force Theory^9^ which is useful against adverse flow.

For microsurgery, recent developments in microrobotics have shown revolutionary potential to improve minimally invasive surgical procedures through highly controllable and precise manipulation, which bring intraoperative and postoperative benefits^8^,^10^,^11^,^12^,^13^,^14^,^15^,^16^,^17^,^18^,^19^,^20^,^21^,^22^. Microrobots have been tailored to move and work in specific homogenous environments, as well as completing specific tasks such as on-surface transportation^23^,^24^,^25^, tissue incision^26^, puncture of retinal veins^27^, and cell scaffolding^22^. However, a reconfigurable system which can travel through various environments and then change configurations to accomplish a variety of tasks would greatly advance microsurgery applications.

Although many types of microrobots have been successfully created, including flexible swimmers^28^,^29^, biological robots^23^,^30^, and chemical propellers^31^,^32^,^33^,^34^, we focus on those actuated and controlled by magnetic fields, which is considered to be the most effective method since magnetic fields permeate over long ranges with minimal health effects and easily transmit large amounts of power^35^,^36^. The most common variety of magnetically-actuated microrobot are helical and chiral^37^,^38^,^39^,^40^,^41^,^42^,^43^. For modular microrobotics, Tottori *et al.* have previously introduced a method to assemble and disassemble helical microswimmers into different configurations using magnetic force and hydrodynamic interactions^44^. However, although their system could dynamically reconfigure size and shape, the underlying helical geometry, while effective for swimming, is not ideal for modularity as the maximum number of assembled swimmers is three, reducing versatility.

In contrast, the modular microrobots we use for this work utilize magnetic beads as the basic building blocks, allowing a much wider range of module and assembled geometries than helical microswimmers. Much like snake-like macroscale modular robots^45^, microscale bead-based modules enable high versatility at low cost, and furthermore synergize well with the particulate drug delivery paradigm. Our modular microswimmers consist of magnetic microbeads assembled by magnetic attraction. The use of particle-based modules is enabled by our previous work on achiral, particle-based microswimmers^46^,^47^,^48^,^49^ which showed that bodies constructed of more than 3 beads can swim. We demonstrate directed assembly of swimmers with other single- or multiple-bead modules, elucidate the physical mechanism of assembly, and use the mechanism to improve assembly efficiency, culminating in a demonstration of modular assembly and disassembly. Finally, to indicate how modular assembly can lead to swimmers capable of different behavior, we investigate how the swimming properties of assembled swimmers vary with size.

## Results

### Experimental observation of disassembly and assembly

We placed a sample of the microswimmers (structures with 3 or more beads) with nonmotile module units (single beads) inside a polydimethylsiloxane (PDMS) chamber (3 mm diameter, 2 mm height). The chamber was sealed to prevent evaporation and to minimize flow, then placed inside approximate Helmholtz coils (see Methods Summary) for experiments. Our previous work showed that upon rotation by an external torque, magnetic structures constructed of three or more beads can be controllably propelled along the rotation axis. We propel swimmers constructed of magnetic microbeads (4.35 μm in diameter, Spherotech) towards additional beads or groups of beads for assembly.

**Figure 1:**
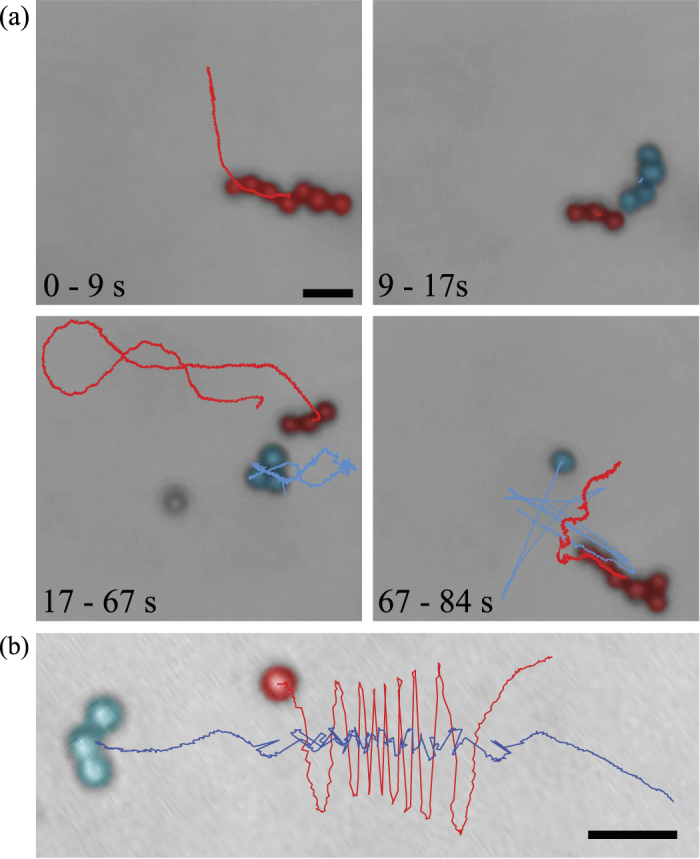
Assembly and disassembly for modular microrobotics. (**a**) Experimental demonstration of assembly and disassembly by manipulation of the control inputs (field strength and rotation frequency). (**b**) During a failure to assemble, the particles orbit around the microswimmer due to hydrodynamic force.

In a representative example shown in Fig. 1(a) [also see Supplementary Movie 1], we observed a 7-bead swimming microswimmer rotating at 15 Hz (0–9 s) transformed into different shapes and size through disassembly and assembly. To disassemble the microswimmer, we increased the rotation frequency (10–15 s). The hydrodynamic stress on the swimmer due to the high rotation frequency physically deformed the swimmer by creating a twisting effect [see Supplementary Movie 1] which led to disassembly at 16s when the swimmer broke into a 3-bead and 4-bead swimmer. The 3-bead swimmer demonstrated swimming at 15 Hz rotation while the 4-bead structure remained stationary (18–65 s). Cheang *et al.* provided a detailed explanation on why one structure can swimming while the other does not^47^. At 65–70 s, the 3-bead swimmer then approached the 4-bead structure for assembly, however, one of the beads from the 4-bead structure detached, resulting in a final 6-bead assembled structure. From 71–84 s, the 6-bead swimmer demonstrated swimming at 15 Hz rotation.

Not every approach leads to successful assembly. Figure 1(b) illustrates a single bead orbiting around an approaching microswimmer at 20 Hz without mechanical contact [see Supplementary Movie 2]. For the 3-bead microswimmers in this paper, most of the approaches at rotation rates higher than 20 Hz resulted in failure.

### Hydrodynamic and magnetic interactions for assembly

Although the observations above demonstrate assembly and disassembly, it is evident that the processes are not robustly controlled. To understand the mechanism of assembly and to improve its effectiveness, we developed a minimal physical model of the process.

**Figure 2:**
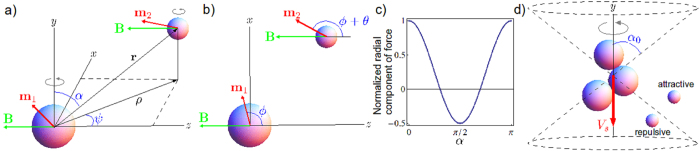
Schematic for assembly model. (**a**) Schematic for magnetic interaction between two magnetic beads with dipole **m**_1,2_ rotated by magnetic field **B**. (**b**) Top view, showing lag angle *θ*. (**c**) Spatial dependence of normalized radial force on angle *α* describes (**d**) cones of repulsion in front and behind swimmer, and attractive region beside swimmer.

We treat the magnetic interaction as the force between two spheres in a viscous fluid with permanent magnetic dipoles rotated by an external magnetic field (schematic in Fig. 2). The magnetic force between two dipoles with moments **m**_1,2_ separated by displacement **r** is


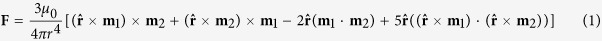


In the absence of interactions, each sphere rotates due to the external field. For rotation rates below step-out, each sphere will rotate at the same rate as the field with a fixed lag angle between each dipole and the field determined by equating the magnetic torque (***m *****× *****B***, where *m* is magnetic moment and *B* is magnetic field) and the rotational drag of the sphere (*8πμr*^*3*^***ω***). Therefore the lag angle *θ* in Fig. 2(b) is constant while *φ* steadily increases. Note that upon adding magnetic interaction between two rotating spheres, the rotation axis and dynamics are not strongly altered: dipole fields decay quickly with separation, and for the typical moments of our beads (≈10^−15^ J/T) dipole fields only approach the magnitude of our external fields (≈1 mT) at distances <<1 μm. Nonetheless, even small non zero radial attraction or repulsion (via equation (1)) can ultimately lead to approach and successful assembly.

Experimentally, it is observed that swimmers rotate quickly compared to changes in position; hence, it is appropriate to average the magnetic force over the angle *φ*. The result of such averaging is a radial component of magnetic force proportional to cos*θ*(1 + 3cos2*α*)/*r*^4^, where *α* is the angle between the displacement vector and rotation axis (Fig. 2(a)), which is also in the direction of average swimming. Since the difference in lag angles (*θ*) is always less than π/2, the radial component of the magnetic force (Fig. 2(c)) is positive or repulsive at small and large *α* (in front or behind the swimmer) and negative or attractive for *α* in a range around π/2 (towards the side of the swimmer). Thus, there are cones of repulsive magnetic force in front of and behind the swimmer and a zone of attraction beside the swimmer (Fig. 2(d)). This simplified magnetic model suggests that during assembly, as a swimmer swims towards a module, it is in the repulsive region (*α* < *α*_*0*_) and gets pushed off axis. Only when the swimmer moves beside the module can the module get attracted to and assemble with the swimmer.

Turning towards hydrodynamic interactions, the simplest model of the hydrodynamics is to treat the module as advected by the swimmer's velocity field, so that it behaves as a Lagrangian tracer particle. To produce swimming, we model the swimmer as a rigid three-sphere assembly with a permanent magnetic dipole (***m***). We calculate the mobility matrix of the swimmer using the method of regularized Stokeslets^50^. The mobility matrix relates the instantaneous velocity and angular velocity to the external magnetic torque (***N*** = ***m *****× *****B***); for homogeneous fields there is no magnetic force on a dipole. As in our previous work, we find a steadily rotating solution to the dynamics when the angular velocity is equal to the rotation of the field, which requires a specific orientation of the swimmer relative to the rotation axis and magnetic field^46^,^49^. Once the orientation of the swimmer is known, the total torque, linear velocity, and angular velocity of the swimmer are determined and hence the velocity field around the swimmer can be calculated from the method of regularized Stokeslets.

**Figure 3:**
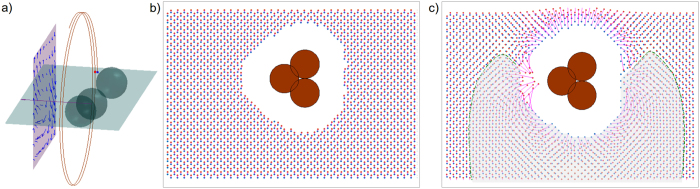
Poincare return map. (**a**) Velocity field on one vertical plane and lagrangian particle trajectory in swimmer frame. Poincare return map reveals movement of bead after one orbit around swimmer, mapping an initial position on horizontal plane (blue point) to the second intersection of its trajectory with the plane (red point). (**b**) Poincare return map of lagrangian particle trajectories for swimmer moving downwards. Initial positions (blue) are mapped to final positions (red). (**c**) Poincare return map when magnetic interactions are included. The shaded area is the area of attraction for particles in front of swimmer.

Without magnetic interactions, integrating the velocity field around a rotating swimmer yields the trajectory of a nonswimming module. To interpret trajectories, we compute a Poincare return map that reveals where a particle moves after one orbit around the swimmer (Fig. 3(a)). The map takes a point on the horizontal plane in the swimmer frame which contains the rotation axis, and maps it to the second intersection of its trajectory with the plane, i.e., its position after a full orbit around the swimmer. Examining the map of all points of the plane (Fig. 3(b)) yields insight into the dynamics; for example, long-term behavior of particles can be deduced from repeated applications of the map. Notably, without magnetic interactions, there is no strong tendency towards attraction or repulsion; rather particles get advected past the swimmer (upwards in the Fig. 3) due to the swimming flow.

Magnetic and hydrodynamic interactions can be combined by altering the instantaneous velocity of the module in the above by the Stokes drag velocity (v = **F**/(6*πμα*_2_) resulting from the magnetic interaction force, which alters the time-integrated trajectories and hence Poincare return map (see Fig. 3(c) for an example). With the addition of magnetic interactions, the Poincare map reveals that (1) there is a stagnation region in front of (below in the Fig. 3) the swimmer magnetic repulsion pushes the module away, leading to difficulty in approach; and (2) all particles in an attraction zone surrounding the front and sides of the swimmer (shaded and delineated by dashed line in Fig. 3) eventually move towards the side of the swimmer as a result of cumulative magnetic forces, even if they are initially repelled in front of the swimmer.

We used the model to improve the success rate of approaching modules for assembly. The model predicts an attractive zone in front of and beside the swimmer. Assembly will only occur if the swimmer can be steered such that the module enters the attractive zone. One method to place the module in the attractive zone is to steer the swimmer so that the module is in front of the swimmer and then swim towards the module. This method requires a relatively small approach angle *α*. Previously, we steered the swimmer to approach the module head-on. The model suggests that head-on approach may be slower due to the stagnation region; instead, a slightly off-center approach may be better. In addition, the model determines how far off-center the approach can be: the range of allowable module placements can be quantified by the width of the attraction region at the bottom of Fig. 3(c), which we call *Δx*. We varied the frequency of rotation in our model to investigate the frequency-dependence of the size of the attraction region and *Δx*. Although there was not a one-to-one correlation due to the fact that the rotation axis of the swimmer changes as frequency changes^50^, in general increasing the frequency decreased both the size of the attraction region and *Δx*. A second method of assembly is, instead of steering so that the module approaches at a small approach angle, to swim near the module, and then turn suddenly by a large angle so that the module is beside the swimmer, i.e. the module enters the side lobes of the attraction region.

### Qualitative investigation of assembly

**Figure 4:**
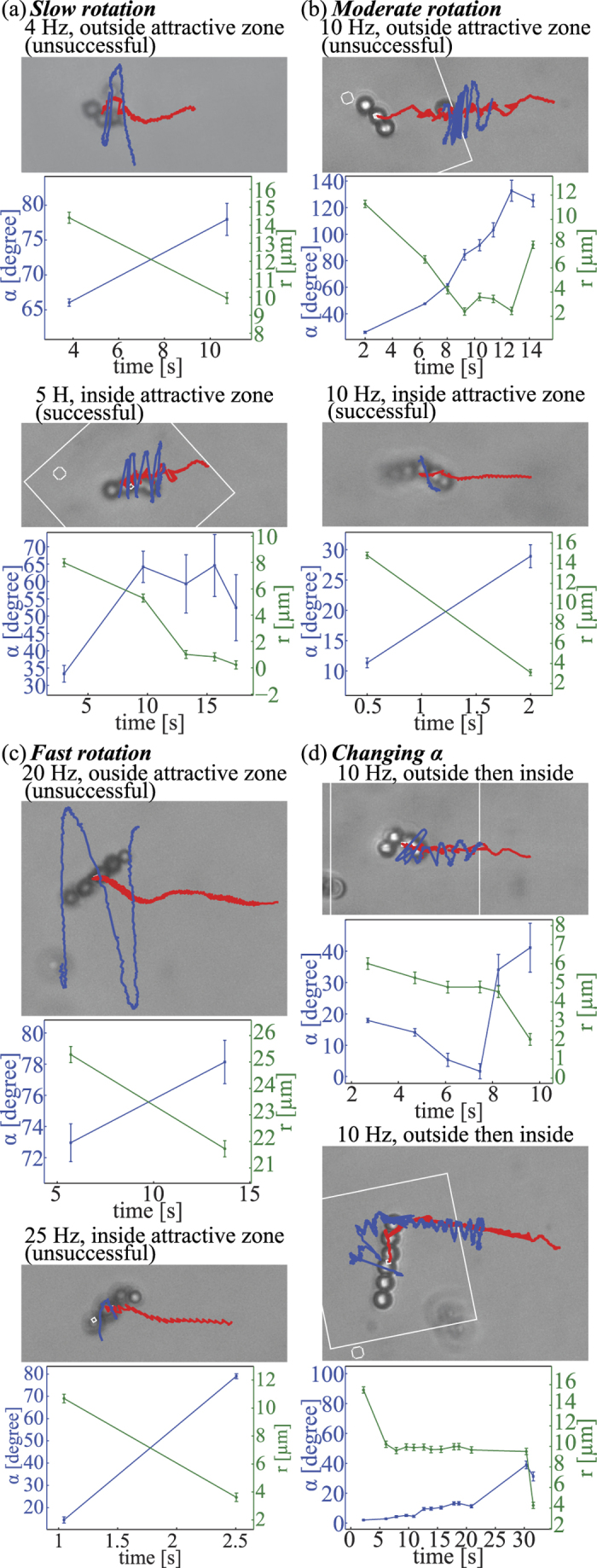
Qualitative experiments of assembly. (**a**) Assembly under low rotation rate (4–5 Hz). The large attractive zone as a result of the slow rotation yields unsuccessful assembly when the orbital distance is very large (top) and successful assembly when the orbital distance is smaller (bottom). (**b**) Assembly under moderate rotation rate (10 Hz). The small attractive zone as a result of the increased rotation rate yields unsuccessful assembly when the orbital distance is very small (top) and successful assembly when the swimmer approaches the single bead at a very small *α* (bottom). (**c**) Assembly under fast rotation rate (20–25 Hz). The attractive zone becomes very small due of the fast rotation rate which results in the single particle being repelled radial causing a very large orbital distance and failure of assembly (top). Successful assembly was possible when the swimmer approach the single bead at a very small *α*, similar to (**b**) (bottom). (**d**) Initial *α* was small resulting in very small orbital distances and trapping in magnetohydrodynamic stagnation region in front of swimmer. When *α* was increased by steering the swimmer to different direction, assembly became possible.

We performed a set of experiments (Fig. 4) to corroborate the predictions of our physical model for assembly and to improve the assembly process. During approach to a single bead, we measured the angle α between the swimmer rotation axis and the displacement vector ***r*** from the swimmer centroid to the approached bead. Due to 2D images from the recorded videos, we were restricted to measuring *α* and ***r*** once per rotation, when the particle and the swimmer lie in the focal plane. First, we note that in each of these and other observations, during approach the particle orbits around the swimmer as predicted from the velocity fields calculated in our model. Second, we verified the existence of an attractive region. In Fig. 4(a–c), we show pairs of approaches at similar frequencies; in each pair there is one unsuccessful approach and one successful approach. As predicted, for each pair the successful approach starts with smaller *α*, placing the module in the attractive width *Δx*, while the unsuccessful approach has larger *α*. Third, the frequency dependence of the size of the attraction zone can be seen by comparing Fig. 4(a–c): for Fig. 4(a), the width of the attraction zone is between *α* = 33.4° (successful) and *α* = 66° (unsuccessful) for 4–5 Hz rotation, while at the higher frequencies the width of the attraction zone is between *α* = 11.3° (successful) and *α* = 26.2° (unsuccessful) for 10 Hz rotation, or *α* = 14.5 (successful) and *α* = 73° (unsuccessful) for 10–25 Hz rotation. In addition, in Fig. 4(b), there are much smaller orbital distances for the unsuccessful approach than in the unsuccessful approach in Fig. 4(a) as the particle passes the swimmer. All of this evidence supports the prediction that increasing the rotation frequency decreases the size of the attractive zone. Fourth, in the initial portions of Fig. 4(d), we show how a head on approach at very small *α* can be disadvantageous due to the stagnation region predicted by the model. For top case in Fig. 4(d), the microswimmers approached the modules at *α* = 17.9° resulting in the particle being trapped inside the stagnation region; as a result, *α* decreased to 7.5° and ***r*** plateaued at 5 µm until 6.1 s. The same phenomena was observed for the bottom case in Fig. 4(d) where the microswimmers approached the modules at *α* = 2° and r plateaued at 9 µm until 20.8 s. Fifth, starting from the head-on configurations in Fig. 4(d), we demonstrate the second method of assembly suggested by our model: we were able to trigger assembly by a change of α (34.1° at 8.2 s, top; 38.9° at 30.4 s, bottom) placing the module beside the swimmer rather than in front of it. To summarize, we found that slower frequencies and small (but not exactly head-on) approach angles were efficient assembly strategies.

### Demonstration of modular microrobotics

**Figure 5:**
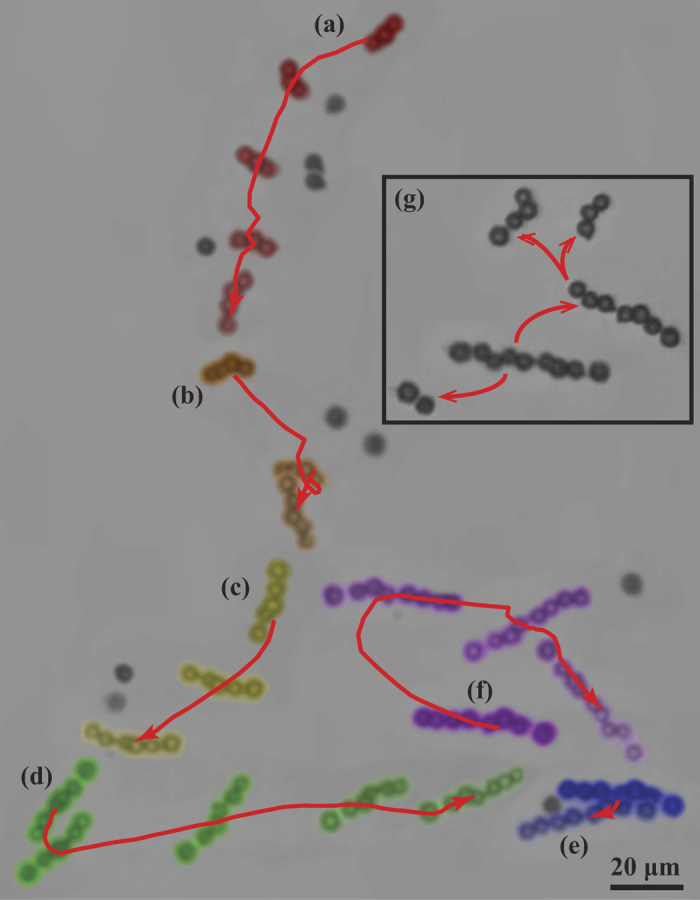
Representative experiment of a modular microrobot. From (a), a 3-bead robotic microswimmer approaches and assemble with a single non-motile bead and transform into a 4-bead microswimmer. From (b–f), the microswimmer continues to approach and combine with single beads, and eventually modulate into a 9-bead microswimmer. At (g), the 9-bead microswimmer breaks into three different microswimmers under high rotation frequency due to increased shear stress leading to structural flexing.

Finally we describe a representative experiment showing the extent of modular capabilities by starting with a microswimmer with minimal number of beads (3 beads) and modulating into a nine-bead swimmer. In accord with the assembly strategy discussed above, at each of the stages involving assembly, the rotation of the microswimmer was decreased in order to promote magnetic assembly. Figure 5 is a collage of the experiment showing 7 stages [see Supplementary Movie 3]. At stage 1, the microswimmer started as a 3- bead microswimmer and assembled with a single non-motile bead to become a 4-bead microswimmer. Likewise from stage 2 to 6, the microswimmer continued to assemble with nearby single beads until it formed a 9-bead microswimmer. At stage 7, a high rotation frequency was introduced which sequentially breaks the microswimmer into three entities: a 2-bead structure, a 3-bead microswimmer, and a 4-beads microswimmer. The 3-bead and 4-bead microswimmers swim controllably, whereas the movement of the 2-bead is not controlled hence we speculate to be driven by neighboring flow fields.

### Assembled swimmers of various sizes have varying swimming behavior

**Figure 6:**
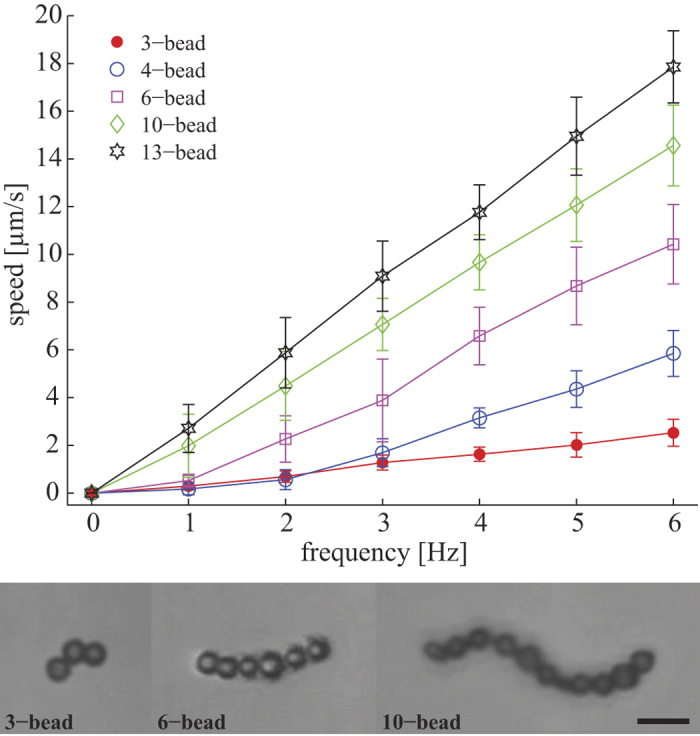
Characterization of swimming capability for microswimmer at various lengths. Swimming speed plotted as a function of rotation frequency for magnetic swimmers with different number of beads. This highlights the increase in swimming power as the size and aspect ratio of the swimmers increase.

Modular microrobotics is most useful if swimmers of different geometries can be constructed with varying abilities to navigate diverse environments or to perform diverse tasks. We have demonstrated modular assembly and disassembly, but our modular microrobotic system has not been optimized for accomplishing such diverse tasks. However, to suggest the potential of a modular approach, we investigate whether the swimmers of different sizes that we create through our modular microrobotics display different behavior. The particle based microswimmers have different configurations which we refer to as 3-bead, 4-bead, 5-bead, etc. While the minimal geometry requirement for low Reynolds number swimming is three beads^46^, microswimmers with more than three beads also demonstrated swimming capability. Usually, dipole-dipole interactions lead to linear assembly^51^, but in the context of our modular system, nonlinear geometries can easily be generated, as seen in Fig. 6, which shows representative examples of three, eight, and thirteen bead microswimmers. This is in fact advantageous since perfectly straight structures cannot generate propulsion upon rotation. To show that different modular configurations have different swimming characteristics, we statistically measured the swimming speed of microswimmers with different body lengths. Microswimmers with more beads tend to swim faster, as shown in Fig. 6. Data for each configuration from Fig. 6 was taken from multiple microswimmers; the three bead swimmers rotating at 6 Hz have an average velocity of 2.53 ± 0.56 μm/s and the eleven bead swimmers have an average velocity of 17.85 ± 1.51 μm/s (see Supplementary Movie 4). The standard deviations are generally larger for microswimmers with more beads due to geometrical reasons, since each additional bead adds to geometrical variability.

## Discussion and Conclusions

We have demonstrated that magnetic microbeads can be used as modular units to create dynamically reconfigurable microswimmers. Versatile modularity is enabled by the ability of generic geometries to be propelled by a rotating magnetic field. Both assembly and disassembly can be achieved. We developed a mechanistic physical model incorporating magnetic and hydrodynamic interactions to understand the assembly process, and used it to find improved assembly strategies, enabling controlled assembly from 3-bead to 9-bead microrobotic systems. Finally, we showed that different configurations have different swimming properties. While we investigated how swimming speed depends on the number of beads in assembled swimmers, for the applications of drug delivery and minimally invasive microsurgery future work remains to demonstrate that different assembled configurations can achieve navigation through various *in vivo* environments, and can be constructed to accomplish different tasks during operative procedures. We believe that the mechanistic insight into the assembly process we discussed in the manuscript will greatly aid future efforts at developing configurations capable of achieving these crucial abilities.

## Methods

### Magnetic control system

To generate the rotating magnetic field which actuates the achiral microswimmers, we used a control system consisting of three pairs of electromagnetic coils arranged in an approximate Helmholtz configuration, three Kepco power supplies (BOP 20–5 M), a National Instruments data acquisition (DAQ) system, a computer, an inverted microscope (Leica DM IRB), and a high speed camera (FASTCAM SA3). Through the use of a DAQ system, the power supplies generate sinusoidal outputs to the coils to create a rotating magnetic field. The high speed camera provides visual feedback and records videos at high frame rates (60–100 fps). The computer is used as interface for the camera and the DAQ system. The three pairs of coils are designed to exert torque on the swimmer without introducing translational force by creating a spatially uniform magnetic field with any specified time-dependent magnitude and direction in a 2 mm × 2 mm × 2 mm region. Experiments take place with swimmers immersed in distilled water (viscosity of 1 mPa∙s) in a 3 mm × 3 mm × 2 mm (L × W × H) polydimethylsiloxane (PDMS) chamber, sealed to minimize fluid flow and evaporation, and placed at the center of the Helmholtz coil system mounted on the microscope.

The coil system is designed to exert constant torque on the microswimmer without introducing translational force. The coils are arranged in a slightly different configuration than that of the normal Helmholtz coil. Conventionally, Helmholtz coil restrict the distance between two coils of the same size to be the radius of the coils. Given the space constraint of our microscope, our configuration was designed to optimize the magnetic field profile in order to create a near constant region at the center of the coils. In this study, the distance between the coils is equal to the outer diameter of the coils plus the thickness for the coil, creating a cube-like configuration for the 3D coil system.

Computer simulation and direct measurements from a tesla meter were used to model the magnetic field generated from the 3D coil system. With 1 Amp of current passing through two pairs of coils, the simulation result yielded a value of 5.06 mT at the center of the field, which matches the experimentally measured value of approximately 5 mT. We also investigated the field profile and demonstrated the ability of the coil system to generate a near-constant magnetic field at the center region approximately within a 2 mm diameter. The size of this region approximately matches the size of the area where experiments take place. Given the size of the microswimmers being tested, the 2 mm region provides sufficient space needed for experimentation.

### Steering microswimmers using magnetic fields

The magnetic field strength (mT), rotational direction of the magnetic bead (CW or CCW), and rotational frequency (Hz) of the field generated by the coils can be controlled through LabVIEW. The effective magnetic field (***B***) is the vector resultant of the *x*-, *y*-, and *z*-components. To create a rotation, there must be at least two pairs of electromagnetic coils; for example, one pair in the *y* direction and one pair in the *z* direction create a rotating field in the *yz*-plane. The two pairs generate sinusoidal outputs with 90° phase lag. Using *xy*-planar control, the resultant field can be expressed as^48^


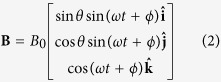


which rotates with angular velocity ***ω*** around the unit vector


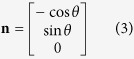


where *B*_*0*_ is the maximum amplitudes of the magnetic field generated by the *x*, *y*, and *z* pair coils respectively, ***ω*** is the rotational frequency of the field, *ϕ* is the phase, *θ* is the direction of rotation, and 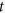 is time. By controlling the field strength of *x*, *y*, and *z* components and the frequency; the frequency of the microswimmer’s rotation and the corresponding mode of motion can be fully controlled.

### Tracking algorithm

A MATLAB tracking algorithm was used for the analysis of the microswimmers’ trajectories. Data taken from experiment are avi video files which can be decompressed in MATLAB and analyzed frame by frame. For each frame, the algorithm processes the image using four main steps: image binarization using grayscale thresholding, structure definition based on pixel continuity, size thresholding to filter out unwanted pixels, and calculation of geometrical centroid. First, in image binarization, a threshold defines a grayscale cutoff value for each pixel and converts the image to binary (black/white). Afterward, the interior of the microswimmer is filled and sequentially labelled as 1, 2, 3, etc. Next, unwanted objects such as debris or out of focus particles are removed using size thresholding which filters out objects bigger and smaller than the microswimmer. At this stage, the only remaining object is the microswimmer and the size of the microswimmer is defined as the number of pixel they occupied. Finally, the geometrical centroid (*x, y*) is calculated.

## Additional Information

**How to cite this article**: Cheang, U. K. *et al.* Versatile microrobotics using simple modular subunits. *Sci. Rep.* **6**, 30472; doi: 10.1038/srep30472 (2016).

## Supplementary Material

Supplementary Information

Supplementary Movie 1

Supplementary Movie 2

Supplementary Movie 3

Supplementary Movie 4
