# Supplementary material for: Versatile microrobotics using simple modular subunits
Source: Sci Rep. 2016 Jul 28;6:30472. doi: 10.1038/srep30472 (PMC4964347; doi:10.1038/srep30472)
Supplement: Supplementary Information [file srep30472-s1.pdf]

Versatile microrobotics using simple modular subunits

U Kei Cheang, Farshad Meshkati, Hoyeon Kim, Kyoungwoo Lee, Henry Chien Fu and Min Jun Kim

Supplementary Movie Legends

Movie S1: Assembly and disassembly for modular microrobotics. The movie shows the use of magnetohydrodynamics for assembly and disassembly.

Movie S2: Magnetic module orbit microswimmer. The movie shows how a module will orbit the microswimmer if assembly is unsuccessful.

Movie S3: Demonstration of modular microrobot. The movie shows a microswimmer transform from three-bead to nine beads, then breaks into two different microswimmers.

Movie S4: Swimming of a 10-bead microswimmer. The movie shows increase in swimming speed of a 10-bead microswimmer as the rotation frequency increased from 1 to 6 Hz.
